# Common polymorphism (81Val>Ile) and rare mutations (257Arg>Ser and 335Ile>Ser) of the *MC3R* gene in obese Polish children and adolescents

**DOI:** 10.1007/s11033-013-2808-8

**Published:** 2013-10-20

**Authors:** J. Cieslak, K. A. Majewska, A. Tomaszewska, B. Skowronska, P. Fichna, M. Switonski

**Affiliations:** 1 Department of Genetics and Animal Breeding, Poznan University of Life Sciences, Poznan, Poland; 2 Department of Clinical Auxology and Pediatric Nursing, Poznan University of Medical Sciences, Poznan, Poland; 3 Department of Pediatric Diabetes and Obesity, Poznan University of Medical Sciences, Poznan, Poland; 4 Present Address: Department of Biochemistry and Molecular Biology, Poznan University of Medical Sciences, Poznan, Poland

**Keywords:** Human, Obesity, *MC3R* gene, Polymorphism, Mutation

## Abstract

The predisposing role to human obesity of the *MC3R* gene polymorphism is controversial. In this report we present the first study focused on the search for the *MC3R* polymorphism in the Polish population. Altogether 257 obese children and adolescents (RBMI>120) and 94 adults, who were never obese or overweight (BMI<25), were studied. For all subjects the entire coding sequence was analyzed by direct DNA sequencing. One common polymorphism (81Val>Ile) and two rare mutations (257Arg>Ser and 335Ile>Ser) were identified. The common polymorphism was widely distributed in the obese and control cohorts, while the mutations were identified in four obese subjects only. In case of the 335Ile>Ser substitution a three-generation family, consisting of 20 members, was also analyzed. It was found that all carriers of the 335Ser mutation were obese, but among non-carriers obese subjects also were found. Our study suggests that the predisposing effect to obesity of the 81Ile polymorphic variant is rather unlikely. With regard to the studied rare mutations we suggest that the 335Ser allele may have a small predisposing effect.

**Electronic supplementary material:**

The online version of this article (doi:10.1007/s11033-013-2808-8) contains supplementary material, which is available to authorized users.

## Introduction

Obesity and overweight constitute an increasingly important medical problem in adults, as well as children and adolescents. In the USA 17 % children and adolescents aged 2–19 years are obese and this incidence rate has tripled since 1980 (http://www.cdc.gov/obesity/childhood/data.html). Data on the prevalence of childhood obesity and overweight in Europe also indicate their alarming levels in some countries [1]. In Poland the prevalence of overweight and obesity, estimated on a group of 17,573 children and adolescents (aged 6–19 years, in the years 2007–2009), was 18.7 and 14.1 % in boys and girls, respectively [2].

It is known that the main type of obesity has a polygenic background, but with a strong influence of environmental factors. The heritability coefficient of this trait ranges from 0.4 to 0.7 [3]. Extensive genomic studies focused on the identification of genes predisposing to obesity revealed presence of more than 50 loci, which polymorphisms are associated with obesity traits [4]. Among them the most important polymorphisms were identified within or in a close vicinity of the *FTO* and *MC4R* genes [3]. These two genes were also studied in Polish obese children and adolescents. It was confirmed that the known *FTO* polymorphism (rs9939609 T>A) is significantly associated with predisposition to obesity [5]. On the other hand, analyses of the entire coding sequence of the *MC4R* gene revealed numerous polymorphisms, but their association with obesity was not evident [6].

The *MCR* gene family consists of five members and two of them (*MC3R* and *MC4R*) are involved in the control of mammalian energy homeostasis and thus their mutations and polymorphisms may predispose to obesity [7, 8]. It should be pointed out that among over 150 missense variants in the *MC4R* gene there are polymorphisms predisposing to polygenic obesity, as well as mutations causing the monogenic type of obesity. On the other hand, the role of the *MC3R* gene polymorphisms and mutations in relation to obesity is still controversial. Among over 20 missense substitutions in the *MC3R* gene two (6Thr>Val and 81Val>Ile) are common polymorphisms and one (335Ile>Ser) is considered as strongly predisposing to obesity [9]. Recently it was shown that the 6Thr>Val polymorphism is not a missense substitution, since it is located in the 5′-flanking region between two ATG start codons, out of which the proximal one is evolutionarily conserved and functional [10]. Since the *MC3R* gene polymorphism has not been studied to date in the Polish population, the aim of our study was to search for polymorphisms in the coding sequence of the single-exon *MC3R* gene in two cohorts; i.e. obese Polish children and adolescents, and adults who had never been obese.

## Materials and methods

### Patients

Altogether 257 patients of the Department of Pediatric Diabetes and Obesity (Poznan University of Medical Sciences, Poznan, Poland) and 94 non-obese adults were included in the study. In the cohort of obese patients there were 130 boys and 127 girls, at an average age of 12.3 years (±3.8), within a range between 4 and 17 years. In this cohort the BMI z-score was calculated for obese children and adolescents, using the LMS method, which presents the distribution of BMI by age and gender, using the formula as follows:

$${\text{z }} = \, \left[ {({\text{ BMI }}/{\text{ M }})^{\text{L}} \, {-}{ 1 }} \right] \, /{\text{ (L }} \times {\text{ S)}},$$

where the values of L, M and S were adequate for Polish population according to the data reported by Kułaga et al. [2].

Additionally, the relative body mass index (RBMI) was calculated, following the formula proposed by Poskitt [11]:

$${\text{RBMI }} = {\text{ (individual BMI }}/{\text{ standard BMI for a given age and gender) }} \times { 1}00\;{\text{(\%)}}.$$

RBMI ratio exceeding 120 % was classified as obesity.

The control group was recruited from healthy adults (both genders) with a body mass index (BMI) below 25, who have never been obese. Additionally hair samples were collected from 19 relatives of an obese-carrier of the 335Ser mutation. The study was approved by the local Bio-Ethics Committee at the Poznan University of Medical Sciences.

## Methods

DNA was isolated from blood samples and hair follicles using the Blood Mini Kit and the Genomic Mini Kit, respectively (A&A Biotechnology, Poland) according to the manufacturer’s instructions. The entire *MC3R* coding sequence (972 bp), starting from the evolutionarily conserved translation start codon, as well as short fragments of the 5′ and 3′-flanking regions (112 and 33 bp, respectively), were PCR amplified as two overlapping amplicons (562 and 639 bp). It should be pointed out that a recent study of Tarnow et al. (2012) revealed that also in human translation starts from the evolutionarily conserved start codon (AUG), which is in opposition to earlier reports. Primer pairs were designed using the Primer3 tool (http://frodo.wi.mit.edu/primer3/) based on NG_012200.1 GenBank sequence. Amplification was conducted in a Biometra T-Gradient thermocycler (Biometra, Germany) using EURx Taq polymerase (EURx, Poland). Primer sequences and PCR amplification conditions are shown in Table S1.

Screening for polymorphisms in all patients and controls was performed by direct sequencing of *MC3R* amplicons with the use of an ABI Prism 3130 automatic sequencer (Applied Biosystems, USA). An association study between 81Val>Ile polymorphism and obesity indices (BMI z-score and RBMI) in obese children and adolescents was conducted using the Kruskal–Wallis test. Statistical analyses were performed in the R statistical package version 2.15.1 (http://www.r-project.org/).

## Results

Altogether 1 common polymorphism (81Val>Ile) and 2 rare mutations (257Arg>Ser and 335Ile>Ser) were found (Table 1). To make our results comparable with previous reports concerning polymorphisms of *MC3R* we decided to follow a common numbering of the codons, starting from the non-conservative upstream translation start site.

**Table 1:** Distribution of the *MC3R* missense variants in obese patients and non-obese control adults

Group	*n*	Genotypes								
		81Val>Ile			257Arg>Ser			335Ile>Ser		
		Val/Val	Val/Ile	Ile/Ile	Arg/Arg	Arg/Ser	Ser/Ser	Ile/Ile	Ile/Ser	Ser/Ser
Obese children and adolescents	257	199	55	3	255	2	0	255	2	0
		(77.4 %)	(21.4 %)	(1.2 %)	(99.2 %)	(0.8 %)	(0.0 %)	(99.2 %)	(0.8 %)	(0.0 %)
Non-obese (control adults)	94	75	19	0	94	0	0	94	0	0
		(79.8 %)	(20.2 %)	(0.00 %)	(100 %)	(0.0 %)	(0.0 %)	(100 %)	(0.00 %)	(0.0 %)

Distribution of the variants at the common polymorphic site (81Val>Ile) was similar in both cohorts (obese children or adolescents, and non-obese adults). The rare missense mutations occurred in four unrelated obese patients who had heterozygous genotypes. The 257Ser variant was carried by two obese patients and the 335Ser variant by another two obese patients. Among carriers of the 257Ser variant one was a heterozygote at codon 81 (Val/Ile), while the other one was a homozygote at this site (Ile/Ile). In case of the carriers of the 335Ser variant, one was a homozygote at codon 81 (Val/Val) and one was a heterozygote (Val/Ile).

A comparison of mean BMI z-score and RBMI between obese patients (excluding 4 carriers of the rare mutations) with different genotypes at codon 81 revealed the lowest BMI z-score and RBMI in Ile/Ile homozygotes (2.0 ± 0.6; 146.3 ± 23.0), while two other genotypes had similar values of both indices (2.34 ± 0.5; 163.1 ± 21.4 in Val/Val and 2.32 ± 0.5; 166.4 ± 26.7 in Val/Ile). However, the observed differences were not significant.

Due to the very small number of obese carriers of the mutations it was not possible to test the significance of the observed differences (Table 2). In case of the 257Ser variant carriers a strongly elevated BMI z-score and RBMI (2.6/197 and 3.2/199) was observed, while carriers of the 335Ser variant had different values of both indices (1.8/135 and 2.5/173).

**Table 2:** BMI z-score/RBMI of patients carrying rare missense mutations in comparison with obese non-carriers of these mutations

Codon/mutation	BMI z-score/RBMI	
	Obese carriers (genotype at codon 81)	Obese non-carriers
257Arg>Ser	Patient 1 (Val/Ile): 2.6/197.0	2.3 ± 0.5/163.6 ± 22.6 (*n* = 239^a^/*n* = 255)
	Patient 2 (Ile/Ile): 3.2/199.0	
	Mean (±SD) for patients 1 and 2: 2.9 ± 0.4/198.0 ± 1.4	
335Ile>Ser	Patient 3 (Val/Val): 2.5/173.0	2.3 ± 0.5/164.0 ± 22.7 (*n* = 239^a^/*n* = 255)
	Patient 4 (Val/Ile): 1.8/135.0	
	Mean (±SD) for patients 3 and 4: 2.2 ± 0.5/154.0 ± 26.9	

*SD* standard deviation

^a^BMI z-score could not be calculated for patients under 6 years

**Fig. 1:**
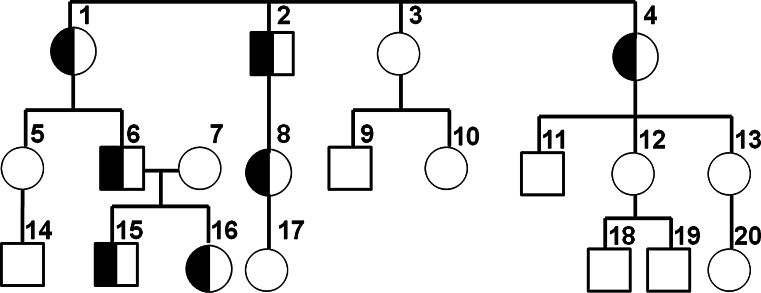
Pedigree of the family in which 335Ser mutation segregated

Additionally, an analysis of obesity indices (BMI, RBMI/BMI z-score) was performed in a family, in which the 335Ser variant segregated. Among the 20 members of this family 8 were obese, 4 were overweight and 8 had normal BMI or RBMI/BMI z-score (Table 3). The 335Ser allele was carried by 7 persons (Fig. 1). All the carriers, except for one woman (#1) who was on a restricted diet after an intestinal surgery, were overweight or obese. With regard to the polymorphism at codon 81 all of the family members were Val/Val homozygotes.

**Table 3:** Obesity indices in a family in which the variant 335Ser variant segregated

Individual no.	Gender	Genotype at codon 335	BMI or RBMI (BMI z-score)
1	F	Ile/Ser	19.9^a^
2	M	Ile/Ser	32.3
3	F	Ile/Ile	44.5
4	F	Ile/Ser	33.9
5	F	Ile/Ile	24.3
6	M	Ile/Ser	37.3
7	F	Ile/Ile	24.9
8	F	Ile/Ser	27.1
9	M	Ile/Ile	28.0
10	F	Ile/Ile	20.5
11	M	Ile/Ile	28.3
12	F	Ile/Ile	20.4
13	F	Ile/Ile	32.1
14	M	Ile/Ile	24.2
15	M	Ile/Ser	36.3
16	F	Ile/Ser	147 (2.24)^b^
17	F	Ile/Ile	35.5
18	M	Ile/Ile	104 (0.30)^b^
19	M	Ile/Ile	85 (−1.35)^b^
20	F	Ile/Ile	28.7
Mean BMI for the carriers, Ile/Ser (±SD)	–	6 subjects (except for one adolescent, #16)	31.1 ± 6.6
Mean BMI for non-carriers, Ile/Ile (±SD)	–	11 subjects (except for two adolescents: #18 and #19)	28.3 ± 7.0

*SD* standard deviation

^a^Patient under restricted diet after intestinal surgery

^b^RBMI (BMI z-score) calculated for adolescents (17, 17 and 14 years old, respectively)

## Discussion

We compared obese children and adolescents (RBMI>120) with healthy non-obese adults as a control in respect to polymorphism of the *MC3R* gene. Since the control subjects never developed obesity during their life it was assumed that their genotype was not predisposing to obesity. For better characteristics of obesity in the investigated cohort we calculated two indices (the BMI z-score and the RBMI), which were used in association analysis.

In this study we searched for polymorphism in the entire coding sequence of the *MC3R* gene. According to the very recent report of Tarnow et al. [10] the *MC3R* gene encodes a protein consisting of 323 amino acids. Previously it was claimed that it consists of 360 amino acids and the translation starts from the non-conserved AUG codon, localized 111 nucleotides upstream of the evolutionary conserved translation initiation site. Since we analyzed the sequence starting from the conserved translation start codon, we did not identify another common polymorphism, known in the literature as 6Thr>Lys, which is in a complete linkage disequilibrium with the 81Val>Ile polymorphism.

The influence of the *MC3R* gene variants on obesity is not evident. There are several reports on the effect of common polymorphism (81Val>Ile) and a majority of them showed a lack of a predisposing effect to obesity (Table 4). In majority of the reports a relatively small number of patients were studied. Only in three studies larger cohorts of obese patients (839, 889 and 1,008) were included. In our study number of obese subjects (257) was similar to majority of the listed reports. Our study also indicates that this polymorphism is rather neutral, which is in agreement with previous functional analyses, indicating the lack of its influence on receptor ligand binding ability [22]. The frequency of the mutation was similar in obese and non-obese cohorts: 0.10 and 0.11, respectively. However, BMI values of obese heterozygotes (Val/Ile) and homozygotes (Ile/Ile) were elevated, but the differences were not significant. There are very few studies concerning the influence of rare mutations (257Arg>Ser and 335Ile>Ser) on obesity and it seems that only the 335Ser allele may predispose to obesity, since it was shown that this variant is responsible for a disturbed ligand binding ability of the MC3R [9]. Our analysis of a three-generation family showed that all carriers of the 335Ser mutation were obese or overweight. It might support the above mentioned suggestion that this mutation slightly predisposes to obesity. Functional in vitro studies of 257Arg>Ser mutation did not confirm its influence on MC3R receptor ligand binding ability [21]. Interestingly, in the previous reports the mutation occurred more frequently in non-obese control patients [13, 21] whereas in our study this variant was found only in 2 obese subjects.

**Table 4:** Three missense substitutions of the *MC3R* gene—their distribution and relation to obesity

Polymorphic sites	Population	Frequency of the mutated alleles in obese subjects (*n*)	Relation of the mutated allele to obesity	Reference
81Val>Ile	Europeans from seven countries: UK, France, Sweden, Spain, Czech Republic, the Netherlands and Denmark	0.08 (184)	No relation, in terms of weight loss after 10-week long dietary intervention	Santos et al. [14]
	Chilean	Non-random sampling (functional study)	No relation	Obregon et al. [15]
	North American	0.12 (889)	No relation	Calton et al. [13]
	Chilean	0.06 (229)	No relation	Obregon et al. [16]
	French	0.08 (308)	No relation	Hani et al. [17]
	North American	0.26 (190)	Ile/Ile homozygotes are predisposed to obesity	Feng et al. [12]
	Asian	0.22 (201)	Increased percentage of body fat content and higher insulin sensitivity in carriers of the Ile allele	Lee et al. [18]
	Italian	0.05 (184)	Lower effect of weight loss program in obese children carrying Ile allele	Santoro et al. [19]
	Belgian	0.07 (1008)	Associated with increased BMI^a^	Zegers et al. [20]
	Polish	0.12 (257)	No relation	This study
257Arg>Ser	French and Italian	0.000 (839)	Not observed, since only two carriers (Arg/Ser) were found among 967 non-obese controls	Mencarelli et al. [21]
	North American	0.001 (889)	Unclear, since mutation occurred in both groups (1 obese and 7 controls)	Calton et al. [13]
	Belgian	0.008 (249)	Four carriers were found among 249 obese patients although initial functional analysis showed no effect	Zegers et al. [22]
	Polish	0.004 (257)	Only two carriers were found among 257 obese patients	This study
335Ile>Ser	French and Italian	0.001 (839)	Two carriers (Ile/Ser) among 839 obese patients and none among 967 non-obese control	Mencarelli et al. [21]
	Italian	0.002 (290)	One carrier (Ile/Ser) among 290 obese subjects. Brother of the carrier was also an obese carrier	Mencarelli et al. [23]
	Polish	0.004 (257)	Only two carriers were found among 257 obese patients	This study

^a^In fact 6Thr>Lys polymorphism was analyzed, which is in a complete linkage disequilibrium with 81Val>Ile

The distribution of common variants at codon 81 was similar in Polish obese and non-obese patients. The frequency of the minor allele (81Ile) in obese children was 0.12 and it was slightly higher when compared with other studies conducted in Europe (Table 4). The highest frequency of the 81Ile allele (above 0.2) was observed in an Asian population. Interestingly, two independent studies based on North American cohorts [12, 13] revealed significantly different frequencies of this variant (0.26 and 0.12, respectively). It was probably due to a different racial composition of the studied obese cohorts. In the first study a relatively high (0.26) prevalence of the 81Ile variant was described in a cohort consisting of 70 % Caucasians, 24 % African Americans and 6 % representing other races [12]. In the African American subjects the frequency was significantly higher than in Caucasians (0.42 vs. 0.11). In contrast, in the latter study, showing a lower frequency of the 81Ile allele (0.12), 85 % of the obese subjects were Caucasians [13].

Knowledge on the distribution of the rare mutations is limited, but the available reports suggest that they occur with a very low frequency (Table 4). Also in this study the variants were found only in obese patients and their frequencies were 0.004. In the control group these mutations were not found, but that group was small (94 adults) and thus it was not possible to compare the frequencies in both cohorts.

Our study confirmed that the *MC3R* gene is significantly less polymorphic than the *MC4R* gene. In our previous report we showed that in the same cohorts of obese and non-obese subjects 6 missense substitutions were found, including 2 novel ones. Among the identified substitutions in the *MC3R* gene the 335Ile>Ser one may be considered as slightly predisposing to obesity.

## Electronic supplementary material

Below is the link to the electronic supplementary material.

Supplementary material 1 (DOCX 13 kb)
