# Supplementary material for: Common polymorphism (81Val>Ile) and rare mutations (257Arg>Ser and 335Ile>Ser) of the MC3R gene in obese Polish children and adolescents
Source: Mol Biol Rep. 2013 Oct 20;40(12):6893–8. doi: 10.1007/s11033-013-2808-8 (PMC3835951; doi:10.1007/s11033-013-2808-8)
Supplement: Supplementary file 1 — Supplementary material 1 (DOCX 13 kb) [file 11033_2013_2808_MOESM1_ESM.docx]

| **Primer sequence** | **PCR product length** | **Annealing**  **temp.** | **Number of cycles** | **Additional information** |
| --- | --- | --- | --- | --- |
| MC3RIF: GGCCAGATTGAATGAGCATC  MC3RIR: AAGATGGTGACGTACCTGTCG | 562 bp | 60ºC | 35 | 5% DMSO supplied |
| MC3RIIF: ATGATCTGCATCTCCCTGGT  MC3RIIR: CCCGACTGATGATCATTTCC | 639 bp | 61ºC | 35 | 5% DMSO supplied |

Table s1.PCR conditions applied for *MC3R* gene amplification.
